# Regulated Acyl-CoA Synthetase Short-Chain Family Member 2 Accumulation during Spermatogenesis

**DOI:** 10.22074/cellj.2020.6306

**Published:** 2019-09-08

**Authors:** Afsaneh Goudarzi, Amir Amiri-Yekta

**Affiliations:** 1 Department of Clinical Biochemistry, School of Medicine, Shahid Beheshti University of Medical Sciences, Tehran, Iran; 2 CNRS UMR 5309; INSERM, U1209; Université Grenoble Alpes; Institute for Advanced Biosciences, 38700 Grenoble, France; 3 Department of Genetics, Reproductive Biomedicine Research Center, Royan Institute for Reproductive Biomedicine, ACECR, Tehran, Iran

**Keywords:** Acyl-CoA Synthetase Short-Chain Family Member 2, Epigenetics, Histone Modifications, Spermatogenesis

## Abstract

**Objective:**

Acyl-CoA synthetase short-chain family member 2 (ACSS2) activity provides a major source of acetyl-CoA to drive histone acetylation. This study aimed to unravel the ACSS2 expression during mouse spermatogenesis, where a dynamic and stage-specific genome-wide histone hyperacetylation occurs before histone eviction.

**Materials and Methods:**

In this experimental study, ACSS2 expression levels during spermatogenesis were verified by Immunodetection. Testis paraffin-embedded sections were used for IHC staining with anti-H4 pan ac and anti-ACSS2. Co-detection of ACSS2 and H4K5ac was performed on testis tubular sections by immunofluorescence. Proteins extracts from fractionated male germ cells were subjected to western-blotting and immunoblot was probed with anti- ACSS2 and anti-actin.

**Results:**

The resulting data showed that the commitment of progenitor cells into meiotic divisions leads to a robust accumulation of ACSS2 in the cell nucleus, especially in pachytene spermatocytes (P). However, ACSS2 protein drastically declines during post-meiotic stages, when a genome-wide histone hyperacetylation is known to occur.

**Conclusion:**

The results of this study are in agreement with the idea that the major function of ACSS2 is to recycle acetate generated after histone deacetylation to regenerate acetyl-CoA which is required to maintain the steady state of histone acetylation. Thus, it is suggested that in spermatogenic cells, nuclear activity of ACSS2 maintains the acetate recycling until histone hyperacetylation, but disappears before the acetylation-dependent histone degradation.

## Introduction

 Acetylation of histones is an important controlled process playing a crucial role in the regulation of gene expression (1). Although several families of histone acetyltransferases (HATs) have thus far been identified and they have been extensively studied, the precise source of acetyl-CoA (as the universal donor of acetyl group used by these enzymes) has just recently attracted attention. Acetyl-CoA used by HATs, comes from different sources due to the activity of cytoplasmic/nuclear enzymes, including ATP citrate lyase (ACLY) and ACSS2 (2-5). 

 Recent investigations have particularly highlighted important role of ACSS2 in general and locus- specific histone acetylation (6). Taking into account the important role of ACSS2 in the control of histone acetylation and tissue-specific gene expression, the question of the expression pattern of its encoding gene is a highly relevant issue. In this regard, spermatogenic cell differentiation is particularly an interesting system, since this differentiation program involves a genome-wide histone hyperacetylation during its late stages, just before the occurrence of a global histoneto- protamine replacement (7). Spermatogenesis is a process generating spermatozoa from progenitor male germ cells, spermatogonia. It involves roughly three different phases including mitotic, meiotic and post- meiotic cells. At the end of mitotic phase, spermatocytes are produced which undergo two meiotic divisions, giving rise to round haploid cells named spermatids. In the post-meiotic phase, these round haploid cells differentiate into elongating/condensing spermatids. This process is accompanied by a genome-wide histone removal and their replacement by small basic proteins, transition proteins (TPs) and protamines, following a series of event coordinated by the histone variant H2A.L.2 (7, 8). 

 Ultimately, the elongating spermatids (EIS) undertake a real metamorphosis to become mature spermatozoa (9, 10). Despite the importance of this genome-wide exchange of histones by small basic proteins and the male genome, the molecular mechanisms underlying histone disappearance have remained poorly explored (9). Previous works have described a wave of genome- wide histone H4 hyperacetylation that occurs in EIS right before histone removal. Recent works suggest that this acetylation signals the recruitment of BRDT (as a double bromodomain containing testis-specific factor) whose first bromodomain is indispensable for the removal of above-mentioned acetylated histones (7, 11). This unique physiological situation, where a global histone hyperacetylation occurs in spermatogenic cells, prompted us to consider spermatogenesis as an interesting and relevant system to monitor the stage- specific ACSS2 protein accumulation. 

## Materials and Methods

###  Chemical and reagents

In this experimental study, the utilized antibodies were as follow: ACSS2 antibody (Cell signaling, USA), anti-Actin (Sigma, Germany) and anti-H4 pan-acetyl (Millipore, Germany). Anti-H4K5ac was kindly provided by Dr. Kimura, Department of Biological Sciences, Tokyo Institute of Technology, Japan. 

###  Protein extraction and Western-Blotting

Total protein content from whole testes and fractionated male germ cells were extracted in 8M urea and they were sonicated using Bioruptor sonication system (Diagenode, Belgium) at 250 J. Protein dosage was assessed using Bradford assay. 

###  Male germ cells fractionation

Male germ cells at different stages of spermatogenesis including pachytene spermatocytes (P), round spermatids (RS) and ElS, were obtained by enrichment on a BSA gradient, as previously described (12).

### Immunofluorescence, histology and immunohistochemistry

Alcohol-formalin-acetic acid-fixed (AFA-Fixed) testes were embedded in paraffin and immunostaining of ACSS2 and H4 pan-acetylation were followed by using immunohistochemistry (IHC) technique, as previously described (11). ACSS2 and histone acetylation were co- detected using anti-ACSS2 and anti-H4K5ac in prepared mouse seminiferous tubules by immunofluorescence assay, as previously described (13). 

### Statistical analysis

The expression levels of ACSS2 in male germ cells were normalized, according to Affymetrix or Illumina standardized processes respectively, and statistics were performed using R software and appropriate script packages. Data are expressed as mean + standard error of mean (SEM), expression levels were compared between the different groups using t tests, and P<0.01 were considered to be statistically significant. 

## Results

### Cell type-specific accumulation of ACSS2 during spermatogenesis

**Fig.1:**
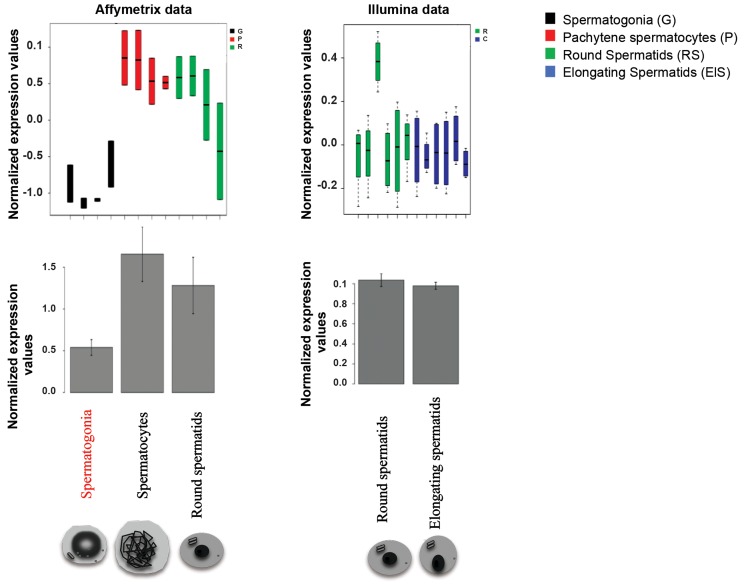
Stage-specific Acyl-CoA synthetase short-chain family member 2 (ACSS2) expression during spermatogenesis. Diagrams show the expression levels of ACSS2 in spermatogonia, spermatocytes, round spermatids and elongating/condensing spermatids. The expression data were obtained from microarray data, available on GEO on the Affymetrix platform GPL1261 (GSE4193, GSE21749, left panel) or the Illumina platform GPL6887 (GSE55767, right panel) (15). A significant increase in the expression level of ACSS2 was observed in meiotic cells (spermatocytes), compared to spermatogonia (P<0.01). Box plots are represented using the default parameters of the function “box plot” in R (black line corresponds to median value and whiskers=1.5 * interquartile range). The histograms represent mean values + standard error of mean (SEM).

**Fig.2:**
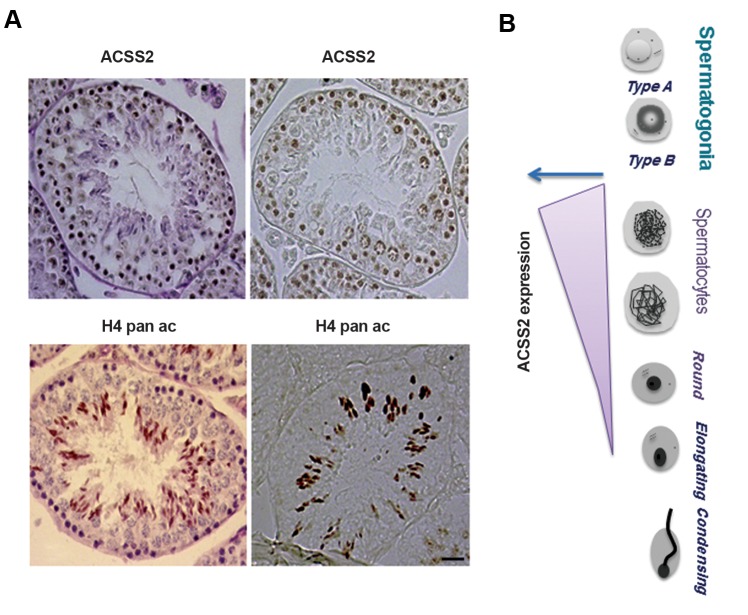
Acyl-CoA synthetase short-chain family member 2 (ACSS2) expression in the mouse testis. A. Immunohistochemistry (IHC) assay was performed on testis paraffin-embedded sections using anti-ACSS2 and H4 pan-acetylated antibodies. The two upper IHC images on the left side represent the ACSS2 signal in sections with and without counter-staining. The two lower IHC images on the left side represent H4 pan-acetylated signal in sections with and without counter-staining (scale bar: 20 μm) and B. The right panel shows ACSS2 expression along spermatogenesis.

To investigate the potential role of ACSS2 in generating essential acetyl-CoArequired for the histone H4 hyperacetylation during spermatid elongation, we focused on the expression level of corresponding protein, during the mouse spermatogenesis. To this end, we took advantage of the previously established stage- specific transcriptomic data (14, 15). This analysis showed a marked increase of ACSS2 expression between spermatogonia and spermatocytes, followed by a slight but not significant decrease in post-meiotic RS and condensing spermatids (Fig .1). Next, to determine the precise pattern of ACSS2 expression during spermatogenesis, we used sections of paraffin- embedded testes and IHC. Figure 2 confirms that ACSS2 could be easily detected in spermatocytes. Rather unexpectedly, this analysis also shows that ACSS2 is not detectable in post-meiotic cells, where histone hyperacetylation occurs (Fig .2). 

**Fig.3:**
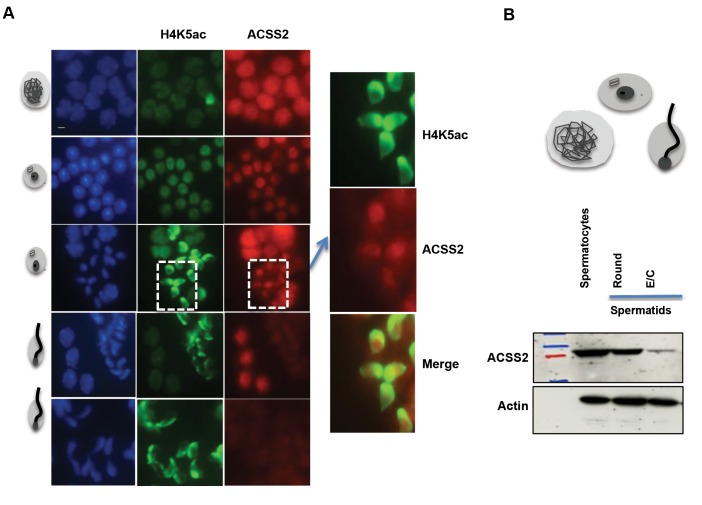
Acyl-CoA synthetase short-chain family member 2 (ACSS2) starts to disappear along with histone removal in post-meiotic cells. **A.** Immunofluorescent co-detection of H4K5ac (in green) and ACSS2 (in red) in mouse male germ cells. DAPI staining was used to visualize cell nuclei (scale bar: 5 μm) and **B. **Extracted total proteins from fractionated mouse male germ cells were used to detect ACSS2 in spermatocytes, round spermatids and elongating/ condensing spermatids fractions using Western-blot.

To better monitor ACSS2 accumulation in spermatogenic cells and more specifically consider its intracellular localization, we used a more sensitive immunodetection of ACSS2 by immunofluorescence. In fact, Figure 3A shows that ACSS2 is robustly accumulated in P, while the protein was predominantly detected in the nucleus. The protein was also detected in post-meiotic cells, especially in EIS, but it did not significantly colocalize with the areas bearing hyperacetylated H4. In contrast, ACSS2 was rather present in nuclear regions where the histones had already been removed, since the regions was devoid of H4K5ac signal corresponding to removed histones and ACSS2 is present in this zone (Fig.3A merged image). Finally, in late elongating/condensing spermatids, ACSS2 was almost undetectable. 

To make sure that the detection of ACSS2 was specific and that the absence of protein in elongating and condensing spermatids was not due to chromatin compaction and the inability of the antibody to detect the protein in situ, we prepared cells enriched at specific stages of spermatogenesis by fractionating spermatogenic cells and performed Western blots, to detect ACSS2 in these fractionated cells.

 The results shown in Figure 3B confirm that ACSS2 is decreased in post-meiotic cells compared to spermatocytes. This also indicates that elongating/ condensing spermatids contain only residual amounts of ACSS2. 

## Discussion

ACSS2 seems to be a major provider of acetyl-CoA for histone acetylation (16). This enzyme could directly use acetate from extracellular or intracellular sources to generate acetyl-CoA (17). One of the important missions of this enzyme is to recycle acetate released after the action of HDACs in the nucleus to feed HATs and maintain the histone acetylation turnover (18). This detailed analysis of ACSS2 expression in parallel with histone acetylation during spermatogenesis actually supports the idea that ACSS2 is, in fact, an enzyme with a major function in acetate recycling to maintain an appropriate amount of acetyl-CoA for histone acetylation. Indeed, it is now known that in elongating/condensing spermatids, the hyperacetylated histones are removed and degraded (7). Hence, under this specific circumstance, there is no more histone to acetylate and therefore no need to recycle acetate to regenerate acetyl-CoA. This situation could explain why ACSS2 is not maintained in the post-meiotic cells, at the stages they undergo histone-to-protamine replacement. Using these observations, we can propose different functions for the two major enzymes generating acetyl-CoA in the cytoplasm and nucleus. These enzymes are ACLY and ACSS2. ACLY uses CoA and citrate to generate acetyl-CoA and oxaloacetate. Although ACLY is capable of efficiently producing acetyl-CoA, it does not play role in recycling the acetate which is released after histone deacetylation. The disappearance of ACSS2 in elongating/condensing spermatids at the time of histone removal supports the idea that acetate recycling is precisely the mission of ACSS2.

## Conclusion

 ACSS2 should be a major actor in maintaining the steady-state of chromatin acetylation, allowing to establish an equilibrium between the action of deacetylases and acetyl-transferases. In elongating/condensing spermatids, hyperacetylated histones are targeted for degradation and hence there is no need to keep active the acetate recycling. This is certainly why ACSS2 disappears in elongating/ condensing spermatids at the time of histone removal.
